# Toward Reliable Synthesis of Superconducting Infinite Layer Nickelate Thin Films by Topochemical Reduction

**DOI:** 10.1002/advs.202309092

**Published:** 2024-04-18

**Authors:** Araceli Gutiérrez‐Llorente, Aravind Raji, Dongxin Zhang, Laurent Divay, Alexandre Gloter, Fernando Gallego, Christophe Galindo, Manuel Bibes, Lucía Iglesias

**Affiliations:** ^1^ Escuela Superior de Ciencias Experimentales y Tecnología Universidad Rey Juan Carlos Madrid 28933 Spain; ^2^ Laboratoire Albert Fert ‐ CNRS, Thales Université Paris Saclay Palaiseau 91767 France; ^3^ Laboratoire de Physique des Solides, CNRS Université Paris Saclay Orsay 91405 France; ^4^ Synchrotron SOLEIL, L'Orme des Merisiers BP 48 St Aubin Gif sur Yvette 91192 France; ^5^ Thales Research & Technology France Palaiseau 91767 France

**Keywords:** nickelates, reduction, superconductivity, thin films, topochemical

## Abstract

Infinite layer (IL) nickelates provide a new route beyond copper oxides to address outstanding questions in the field of unconventional superconductivity. However, their synthesis poses considerable challenges, largely hindering experimental research on this new class of oxide superconductors. That synthesis is achieved in a two‐step process that yields the most thermodynamically stable perovskite phase first, then the IL phase by topotactic reduction, the quality of the starting phase playing a crucial role. Here, a reliable synthesis of superconducting IL  nickelate films is reported after successive topochemical reductions of a parent perovskite phase with nearly optimal stoichiometry. Careful analysis of the transport properties of the incompletely reduced films reveals an improvement in the strange metal behavior of their normal state resistivity over subsequent topochemical reductions, offering insight into the reduction process.

## Graphical abstract

Infinitelayer (IL) nickelate superconductors are synthesized by successive topochemical reductions of the perovskite phase. Careful analysis of the transport properties of the incompletely reduced films reveals an improvement in the strange metal behavior of their normal state resistivity over subsequent reductions. Removal of the apical oxygen anions from the perovskite phase is confirmed through the structural analysis of the IL phase.

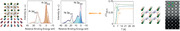

## 1 Introduction

The first observation of superconductivity at relatively high temperature in single‐crystal thin films of infinite layer (IL) NdNiO_2_ upon hole doping was a significant breakthrough.^[^ ^1^ ^]^ Thereafter, superconductivity has also been observed in other families of hole‐doped IL nickelate thin films, such as (Pr, Sr)NiO_2_,^[^ ^2^, ^3^, ^4^ ^]^ (La, Sr)NiO_2_,^[^ ^5^, ^6^ ^]^ (La, Ca)NiO_2_,^[^ ^7^ ^]^ and (Nd, Eu)NiO_2_;^[^ ^8^ ^]^ in reduced Ruddlesden‐Popper Nd_6_Ni_5_O_12_ thin films without chemical doping;^[^ ^9^ ^]^ and in bilayer Ruddlesden–Popper La_3_Ni_2_O_7_ bulk single‐crystals under high pressure.^[^ ^10^ ^]^ Thus, this new class of oxide superconductors provides a new route beyond copper oxides to address outstanding questions in the field of unconventional superconductivity,^[^ ^11^ ^]^ such as the mechanism that causes the electrons to form pairs, unanswered despite decades of intense research activity.^[^ ^12^, ^13^, ^14^, ^15^, ^16^, ^17^ ^]^

Despite an unremarkable critical temperature of around 10 K, that initial observation generated intense interest.^[^ ^18^, ^19^, ^20^, ^21^ ^]^ Part of the reason for that is the discovery of superconducting (SC) nickelates was driven by the decades‐long search of cuprate‐like physics in other strongly correlated metallic oxides. Within this context, Ni‐based compounds with Ni ions arranged in corner‐sharing NiO_4_ square units and ultralow chemical valence (Ni^1 +^ and 3d^9^ configuration) were suggested to exhibit superconductivity due to their electronic and structural similarities with the Cu^2 +^ ions in cuprates.^[^ ^22^ ^]^ However, besides these similarities, very different behavior between LaNiO_2_ and CaCuO_2_ was pointed out early on.^[^ ^23^ ^]^ And, whether there is a universal mechanism that explains superconductivity in both cuprates and nickelates remains an open question.^[^ ^24^, ^25^, ^26^, ^27^, ^28^, ^29^, ^30^, ^31^, ^32^ ^]^

Further progress in the field crucially depends on the synthesis of high quality SC nickelate samples, which can provide reliable experimental data. However, only a few groups worldwide have developed the appropriate expertise to date,^[^ ^1^, ^4^, ^7^, ^8^ ^]^ since the synthesis of these films poses serious challenges.

Epitaxial growth of complex oxides thin films, such as nickelates, is achieved under vacuum at high substrate temperatures to increase surface mobility of adatoms and improve crystallinity of the grown films. At these elevated temperatures, the process yields the most thermodynamically stable phase with octahedral NiO_6_ coordination. Thus, the synthesis of the reduced structure with square‐planar coordination around Ni^1 +^ ions, arranged in infinite layers, should proceed via the subsequent removal of relatively mobile oxygen anions at low temperatures, by means of kinetically controlled reactions that enable the preparation of metastable phases.^[^ ^33^ ^]^

This transformation of the starting complex oxide phases into oxygen‐deficient metastable phases can be triggered by different methods,^[^ ^34^, ^35^, ^36^ ^]^ and is topotactic since it does not involve diffusive rearrangement of the host cations, although lattice parameters and bond lengths change, giving rise to drastic changes in electronic structures. Thus far, in the case of hole‐doped nickelates, the topotactic transition from the perovskite phase into the highly metastable SC IL phase has been accomplished ex situ by low temperature annealing within a sealed glass ampoule using CaH_2_ as the reducing agent,^[^ ^1^, ^2^, ^5^ ^]^ and in situ using an oxygen getter metal layer.^[^ ^8^ ^]^ Under the former topochemical approach, the resulting phase can be tuned by the choice of the metal hydride and by the reaction conditions, in particular, temperature and time.^[^ ^37^, ^38^, ^39^ ^]^ On the one hand, the temperature of the topochemical reaction has to be high enough to bring about the reduction as the activity of metal hydrides in solid state reduction declines at lower temperature. On the other hand, the perovskite framework is more stable at low temperature since an increase in temperature can result in non‐topotactic reactions, if the cations in the resulting metastable phase become mobile, leading to degradation of the sample crystallinity. Furthermore, the crystalline quality of the starting perovskite phase greatly affects the reaction.^[^ ^40^ ^]^ Indeed, decreasing the lattice mismatch (tensile strain) between the parent perovskite phase and the substrate enhances crystallinity of the subsequent reduced phase, even though the increase in the in‐plane lattice parameter upon reduction leads to higher compressive strain for the reduced phase in that case.^[^ ^41^ ^]^

Here, we report the successful synthesis of SC strontium‐doped praseodymium nickelate thin films. Complementary to previous approaches, we study the cation stoichiometry of the starting perovskite films by X‐ray photoelectron spectroscopy (XPS). Furthermore, we carry out a comprehensive study on transport properties of intermediate reduced films, and find an enhancement of the strange metal behavior of the normal state resistivity of incompletely reduced films over subsequent topochemical reductions. Moreover, the removal of apical oxygen anions from the perovskite phase is confirmed through the structural analysis of the IL phase using four dimensional scanning transmission electron microscopy (4D‐STEM).

## 2 Results and Discussion

As a first step, we optimize the growth conditions of the Pr_0.8_Sr_0.2_NiO_3_ parent perovskite films (hereafter, PSNO_3_) with thicknesses between 10 and 13 unit cells (u.c.) by pulsed laser deposition (PLD) on TiO_2_‐terminated (001)‐oriented SrTiO_3_ (STO) substrates. Bulk PrNiO_3_ crystallizes in an orthorhombic structure (space group #62 *Pbnm*, GdFeO_3_‐type) with lattice constants at room temperature of *a* = 5.42 Å, *b* = 5.38 Å, *c* = 7.63 Å (pseudocubic constant *a*_*pc*_ ≈ 3.82 Å).^[^ ^42^ ^]^ Its crystalline structure is not modified by doping with Sr^2+^ although this brings about a contraction of the unit cell, despite the larger effective size of Sr^2 +^ compared to Pr^3 +^.^[^ ^43^ ^]^ Thus, assuming a Poisson ratio of ν = 0.3, a value common to other oxide perovskites,^[^ ^44^ ^]^ the expected out‐of‐plane lattice parameter for epitaxially grown PSNO_3_ films on STO substrates, inducing 2.23% of tensile strain, is ≲ 3.78 Å. This estimate leads to a value of 2θ ≳ 48.04° for the (002)_*pc*_ reflexion in the x‐ray diffraction pattern (using Copper K‐α radiation). Interestingly, that value is in agreement with the threshold experimentally found in the (Nd, Sr)NiO_3_ system, where if the (002) pseudocubic perovskite peak 2θ position is below ≈48°, the subsequently reduced film never exhibits superconductivity.^[^ ^40^ ^]^

We use complementary information from X‐ray Diffraction (XRD) and resistivity as function of temperature, ρ(*T*), to elucidate the optimal growth conditions. Laser fluence has a major impact on the film quality, as expected.^[^ ^45^, ^46^ ^]^ Initially, we have explored laser fluences ranging from 1.2 to 3 J cm^−2^ at temperatures around 600 °C in a strongly oxidizing environment with 0.3–0.4 mbar of O_2_, required to stabilize a Ni^3.2 +^ oxidation state.

**Figure 1:**
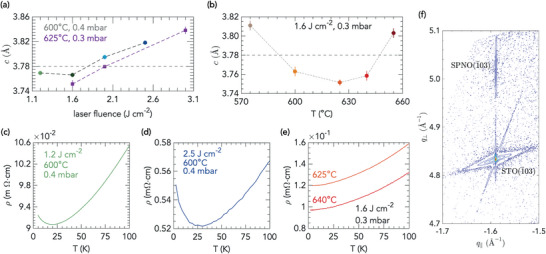
Optimization of growth conditions for PSNO_3_. a) *c*‐axis lattice parameter of PSNO_3_ films as function of laser fluence at various conditions of substrate temperature and oxygen pressure in the PLD chamber during growth. b) *c*‐axis lattice parameter as function of substrate temperature, keeping constant laser fluence at 1.6 Jcm^−2^ and oxygen pressure at 0.3 mbar. The dashed horizontal line in panels (a) and (b) is an estimate of *c* from the bulk lattice constant of PNO_3_ strained to the STO substrate, and doping with Sr is expected to bring about a contraction of the unit cell. c, d, e) Temperature dependence of resistivity, ρ(*T*), in PSNO_3_ films grown at different conditions of laser fluence, substrate temperature and oxygen pressure, whose *c*‐axis lattice parameters are shown in panel (a) or (b). f) High‐resolution RSMs around the $\left(\bar{1}03\right)$ reflection of a PSNO_3_ film under optimized conditions (1.6 J cm^−2^, 640 °C, 0.3 mbar); indices with respect to the pseudocubic unit cell. The ($\bar{1}$03) reflection from the STO substrate is also shown. Error bars in panels (*a*) and (*b*) indicate the 1σ uncertainties of the fits.

As illustrated in **Figure** **1**a, the out‐of‐plane lattice parameter *c* of the PSNO_3_ films shows a minimum for a laser fluence of 1.6 J cm^−2^ for different conditions of oxygen pressure and substrate temperature. The cell expansion observed as the fluence departs from that value is indicative of cation vacancies in the films. Oxygen vacancies can be ruled out as the cause of the increase in lattice parameter, since the higher the oxygen pressure is, the more the unit cell expands. A more detailed exploration of the out‐of‐plane lattice parameter as a function of the substrate temperature at a laser fluence of 1.6 J cm^−2^ in 0.3 mbar of O_2_ is shown in Figure 1b. Under these conditions, *c* lattice constants consistent with a low density of defects are found for substrate temperatures in the range from 600 to 640°C. We confirm the superior quality of the films grown at 1.6 J cm^−2^ by electrical transport measurements, as depicted in Figure 1c, d, e.

Indeed, a resistivity upturn at low temperature is observed at fluences of 1.2 or 2.5 J cm^−2^, with minima at around 19 and 30 K, respectively, Figure 1c, d, that may be attributed to localization phenomena either through weak localization or by electron–electron enhanced interactions,^[^ ^47^, ^48^ ^]^ while a metallic state is observed down to 2 K in films grown under 1.6 J cm^−2^, 0.3 mbar, and 625 or 640°C, Figure 1e. Moreover, reciprocal space maps (RSM) around the asymmetric $\left(\bar{1}03\right)$ reflection of PSNO_3_ films grown under the latter conditions demonstrates that the films are fully strained to the STO substrate, as depicted in Figure 1f. See also Figure S1 (Supporting Information) on the optimal growth conditions. Detailed analysis of the resistivity of those films in the low temperature region in the framework of a model that includes quantum corrections is presented in Figure S2 (Supporting Information).

**Figure 2:**
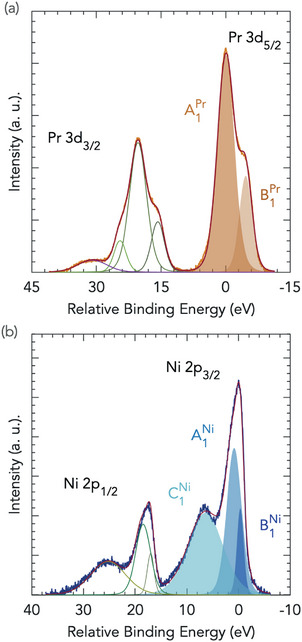
Cation stoichiometry of PSNO_3_ films by XPS. Peak models used for the quantification of the [Pr]/[Ni] ratio. The components (a) brown areas, Pr; b) blue areas, Ni) and total fit envelope are shown.

We probed cation stoichiometry of the PSNO_3_ films by means of X‐ray XPS. Elemental composition of the films and assignments of the peaks in a XPS survey spectrum are plotted in Figure S3 (Supporting Information). Quantitative XPS for the determination of the [Pr]/[Ni] ratio was derived from the area under the core‐level and satellite peaks, Pr 3*d* and Ni 2*p*, as shown in **Figure** **2**, and relative sensitivity factors derived from photoionization cross‐sections by Scofield.^[^ ^49^ ^]^. The precise details regarding the quantification of cation stoichiometry of PSNO_3_ films are given in Section S2 and Figure S4 (Supporting Information).

**Figure 3:**
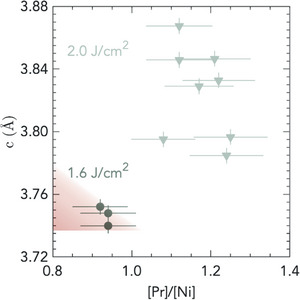
*c*‐axis lattice parameter of PSNO_3_ films as a function of cation stoichiometry. *c* lattice parameter versus [Pr]/[Ni] ratio of PSNO_3_ films grown at laser fluence 1.6 J cm^−2^, 0.3 mbar and substrate temperatures of 625 °C or 640 °C or under non optimal growth conditions of laser fluence 2 J cm^−2^ for different series of films, extracted from the fitted XPS spectra. See Figure S5 (Supplementary Information) on different growth conditions. A laser fluence of 1.2 J cm^−2^ gives rise to *c* = 3.77 Å and [Pr]/[Ni] = 1.07. Error bars of 15% are applied to the [Pr]/[Ni ratio, which is the expected accuracy of XPS quantification for transition metal oxides.^[^ ^50^ ^]^ The red shaded area represents the target region within the error for optimized films.

**Figure** **3** shows *c*‐axis lattice parameter as a function of [Pr]/[Ni] ratio of PSNO_3_ films grown under optimal growth conditions of substrate temperature and oxygen pressure and a laser fluence of 1.6 J cm^−2^, or at a laser fluence of 2 J cm^−2^. Given the complex satellite structure of the XPS spectra, one may expect ≈15% accuracy for the XPS quantification.^[^ ^50^ ^]^ Within these limits of accuracy, nearly stoichiometric films are obtained for an optimal laser fluence of 1.6 J cm^−2^, while films grown at a laser fluence of 2 Jcm^−2^ present a dramatic increase in the [Pr]/[Ni] ratio, with strong deviations from the expected film stoichiometry. Optimal cation stoichiometry yields the lowest unit‐cell volume, in agreement with previous results about films grown by molecular beam epitaxy.^[^ ^51^ ^]^ Figure S5 (Supporting Information) shows in detail the dependence of [Pr]/[Ni] ratio on oxygen pressure and substrate temperature at 2 J cm^−2^. We also assessed the [Pr]/[Sr] ratio from Pr 3*d*_5/2_ and Sr 3*d*_5/2_, and found values close to [Pr]/[Sr] ≈ 4, indicating no strong deviations relative to the nominal hole‐doping levels across different growth conditions, as depicted in Figure S6 (Supporting Information).

**Figure 4:**
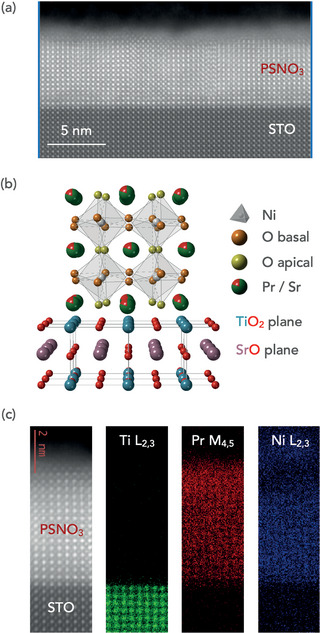
Structure of a PSNO_3_ film grown under optimized conditions on an STO substrate. a) HAADF‐STEM image of a PSNO_3_ film grown at a laser fluence of 1.6 J cm^−2^, 0.3 mbar, and 625 °C. b) Schematic of the structural model of the films grown on STO substrate. c) Atomic‐resolution HAADF‐STEM image and simultaneously recorded elemental EELS maps of Ti L_2, 3_, Pr M_4, 5_ and Ni L_2, 3_ edges.

To provide further structural characterization of the grown films and visualize possible defects of the atomic lattice, cross‐sectional scanning transmission electron microscopy (STEM) imaging was carried out. **Figure** **4**a depicts a high‐angle annular dark‐field STEM (HAADF‐STEM) image of an optimized PSNO_3_ film over a wide area of the film with very few vertical Ruddlesden–Popper faults (the structural model is illustrated in Figure 4b). Electron energy‐loss spectroscopy (EELS) map analysis indicate a uniform distribution of Ni and Pr across the film and an abrupt interface with the STO substrate, Figure 4c).

Having accomplished the growth of PSNO_3_ films with close to optimal stoichiometry and minimal defect densities, we now focus on the study of reduced samples Pr_0.8_Sr_0.2_NiO_2_ (hereafter, PSNO_2_). The as‐grown 5 × 5 mm^2^ films were cut into two pieces with lateral dimensions of 2.5 × 5 mm^2^ or four pieces of 2.5 × 2.5 mm^2^, approximately, before carrying out the hydride reduction. A same piece from the as‐grown sample is repeatedly reduced. Between annealings, the sealed, evacuated glass tube that contains the reducing agent (CaH_2_, physically separated from the sample) is unsealed to measure the sample, and, hence, a new tube is evacuated and sealed to carry out the next step.

**Figure 5:**
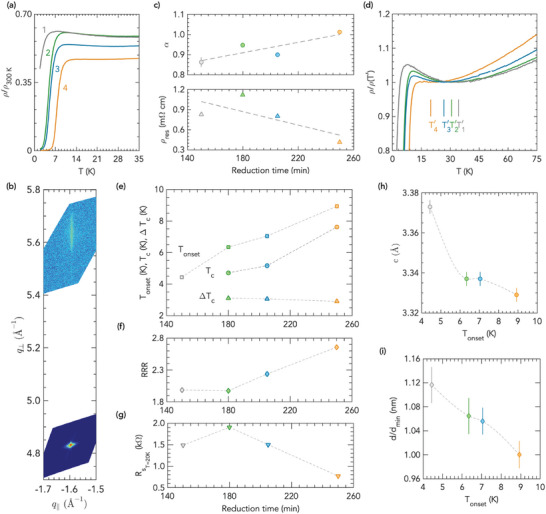
Topotactic phase transition over subsequent topochemical reductions: Electrical transport, superconducting properties, and structural characterization of reduced films. a) Temperature dependent resistivity normalised to its room temperature value of a representative PSNO_2_ film after successive reduction processes carried out at 260°C for periods of (1) 150 min, (2) 30 min, (3) 25 min, and (4) 45 min. b) RSM of the complete reduced IL film around the $\left(\bar{1}03\right)$ reflection. c) Exponent α (top) and residual resistance ρ_*res*_ (bottom) in ρ(*T*) = ρ_*res*_ + *AT* ^α^. d) Evolution of resistivity, normalized to its local minimum value, depicting upturns at temperatures above the onset of the SC transition. e) Evolution of SC properties as a function of reduction time: onset SC transition temperature, *T*_*onset*_, defined as the intersection of linear extrapolations from the normal state and the superconducting transition region; critical temperature, *T*_*c*_, 50% of normal state at 20 K; and, transition width, Δ*T*_*c*_, 90%–10% of normal state at 20 K. f) Residual Resistivity Ratio (RRR), defined as the resistivity at 300 K divided by the extrapolation of the temperature‐linear resistivity to 0 K. g) Sheet resistance *R*_*s*_ at 20 K. h) *c*‐axis lattice parameter and i) thickness of the PSNO_2_ films normalized to the maximum reduced thickness, against *T*_*onset*_. Error bars denote the 95% confidence interval for each fitting (panels *c*, *h* and *i*), and are sometimes smaller than the marker size. See also Experimental section for details on lattice parameters calculation. The dashed lines in all the panels are a guide to the eye.

**Figure** **5**a shows temperature dependent resistivity normalized to its room temperature value (to remove the influence of geometric factors) of a SC PSNO_2_ film over successive reductions carried out at 260°C for periods of 150 min (1), 30 min (2), 25 min (3), and 45 min (4), (see Figure S7, Supporting Information for the extended range 0 – 300 K). Although superconductivity arises after the first reduction, the zero‐resistance state is achieved as a result of further heating periods. The absolute value of resistivity is ρ(20 *K*) ≈ 0.4mΩ cm for the fully reduced sample (Figure S8, Supporting Information), matching the previously published value for SC PSNO_2_ films on STO.^[^ ^3^ ^]^ No diffraction reflections of the parent perovskite phase are detected in the XRD patterns of the reduced samples, and positions of the observed reflections are consistent with the (001) and (002) diffractions of the tetragonal IL phase (Figure S9, Supporting Information). In addition, the RSM in Figure 5b reveals that after the fourth step the reduced phase remains fully strained to the substrate. This is noteworthy because perovskite PSNO_3_ thin films on STO substrates undergo a change from tensile strain to compressive strain as they turn into the IL tetragonal PSNO_2_ phase. Indeed, upon deintercalation of apical oxygen atoms, the in‐plane (out‐of‐plane) lattice parameter of the IL PSNO_2_ phase expands (drastically shrinks) relative to that of the parent perovskite PSNO_3_ phase,^[^ ^37^, ^38^ ^]^ and STO substrates induce a compressive strain (≈−1.2%) on that reduced IL phase.

Resistivity of the SC fully reduced film decreases linearly in *T* from 300 K down to around 60 K, and deviates from linearity below that temperature, likely because of scattering due to disorder, as we discuss below. *T*‐linear normal‐state resistivity has been observed in other families of unconventional superconductors, in particular in the high *T* and low *T* (superconductivity supressed with a magnetic field) normal‐state of cuprates,^[^ ^52^, ^53^ ^]^ and has also been recently found in optimally doped (Nd, Sr)NiO_2_ films with improved crystallinity and reduced disorder synthesised on LSAT substrates.^[^ ^41^ ^]^

We carried out least‐square fitting of the experimental data to ρ(*T*) = ρ_*res*_ + *AT* ^α^ in the range from 300 to 60 K to determine the power law dependence of resistivity (Figure S10, Supporting Information), and found that linearity within this region increases over subsequent reductions, i.e., α → 1: fitted exponents go from α = 0.86 ± 0.02 for the first incomplete reduction to α = 1.01 ± 0.01 for the fully reduced film, as depicted in Figure 5(c, top panel). We also observed that the value of ρ_*res*_, usually taken as a signature of the disorder contribution to the resistivity, tends to lower values throughout the reduction, Figure 5(c, bottom panel). Additional reductions after a fitted exponent α ≈ 1 has been reached do not increase the critical temperature and might deteriorate the film (see Section S7 and Figures S11 and S12, Supporting Information). Moreover, the gradient of linear resistivity in the high‐T range upon complete reduction is $A=2.1\pm 0.05\mu \Omega \;\text{cm}\;K^{-1}$, within the same order of magnitude than $\approx 1.1\mu \Omega \;\text{cm}\;K^{-1}$ empirically found across different materials,^[^ ^54^ ^]^ including highly crystalline (Nd,Sr)NiO_2_ films.^[^ ^41^ ^]^

Proximity to a quantum critical point, Planckian dissipation and existence of a charge gap (Mott insulation) that remains upon doping have been proposed as underlying principles of linear‐in temperature resistivity observed in some metals,^[^ ^55^ ^]^ but there is no widely accepted explanation. To further elucidate the resistive behavior of nickelates superconductors, additional experimental exploration of different manifestations of anomalous behavior in magnetotransport properties of the normal state, such as *H*‐linear magnetoresistance at high field *H*, over a broad doping range, is needed. Nonetheless, the fact that *T*‐linear resistivity is observed in nickelates superconductors that are not doped Mott insulators indicate that Mott insulation of the undoped parent compound is not a necessary ingredient for *T*‐linear resistivity. Furthermore, these results support those recently found in nickelates superconductors with enhanced crystallinity on LSAT substrates.^[^ ^41^ ^]^

Upon further cooling, we find a resistivity upturn that holds between a temperature *T*′, at which ρ(*T*) reaches its local minimum, and the onset of the SC transition, Figure 5d. There is evidence of this behavior for previously reported SC nickelate thin films on STO substrates.^[^ ^1^, ^5^ ^]^ This feature appears to provide additional support for a decrease of disorder in our films throughout the reduction since the temperature *T*′ lowers along the reduction process from 34.3 K after the first step to 26.7 K after the third, and reaches 19.8 K after the fourth, narrowing the temperature range over which the resistivity increases. Also, the upturn gradually smooths out and the resistivity curve flattens, nearly vanishing for the fully reduced sample, whose resistivity exhibits a very weak minimum. Additional transport characterization is shown in Section S8 (Supporting Information).

Figure 5e illustrates the evolution of SC parameters of the reduced sample over successive annealings. Incremental reduction of the film results in a gradual increase of the onset of superconductivity, *T*_*onset*_, and of the critical temperature *T*_*c*_, defined as the temperature at which resistance reaches half the normal state value at 20 K. The highest *T*_*onset*_ ≈ 9 K, which corresponds to a critical temperature of *T*_*c*_ = 7.6 K, and the lowest resistivity ρ(*T*_*c*_) = 0.2mΩ cm (Figure S8, Supporting Information) are found after the whole reduction process. The width of the resistive transition (90%–10% of normal state at 20 K) in the absence of an applied magnetic field, Δ*T*_*c*_, is around 3 K, and slightly decreases throughout the process. No transition widths are given in literature for SC nickelates, and they are difficult to discern from the plots, but widths of the order of 2 or 3 K are usually found in high quality samples of doped cuprates.^[^ ^56^ ^]^

The observed increase in *T*_*c*_ over incremental reduction processes can be attributed to a decrease of disorder, supporting the discussion above. Furthermore, intrinsic inhomogeneity inherent to doping, such as inhomogeneous charge density due to random distribution of dopants and disorder owing to difference in the ionic radius of Pr^3 +^ and Sr^2 +^, can reduce the attainable *T*_*c*_ as it has been reported in cuprates.^[^ ^57^, ^58^ ^]^ Although, on the other hand, nanoscale electronic disorder, studied using scanning tunneling microscopy and spectroscopy, has been found to coexist with high SC transition temperatures in cuprates.^[^ ^59^, ^60^ ^]^ In our results, since the dopant distribution is constant along the reduction process, the increase in *T*_*c*_ suggests an overall decrease of structural defects as the reduction progresses. However, scanning tunneling microscopy experiments are needed on nickelates to reveal the effect of dopant disorder on their local superconducting properties.

The Residual Resistivity Ratio (RRR), which is a measure of the scattering rate of charge carriers by impurities or defects in the films, and is also thought to be indicative of residual apical oxygen atoms in the NiO_6_ octahedra in the reduced phase, is depicted in Figure 5f. As a reference, the RRR in the as‐grown sample was ≈11. In the reduced films, the RRR increases through the overall reduction process, suggesting a progressive decreasing of structural disorder over the orthorhombic to tetragonal transition along with a gradual removal of apical oxygen anions. RR ratios of around 2.8 are achieved, approximately equal to those attained elsewhere.^[^ ^2^, ^3^ ^]^ The significant improvement of the SC transition after the second step does not result in a subsequent increase in the RRR. This may indicate the formation of a restricted SC region in the sample, surrounded by regions that do not superconduct.

As shown in Figure 5g, the normal state sheet resistance measured at 20 K lowers down to *R*_*s*_ ≈ 770 Ω □^−1^ (≈0.030 in *he* ^−2^ units, where *h* is Planck's constant and *e*, elementary charge, taken as an approximative value for the Mott‐Ioffe‐Regel limit in two dimensions). The increase of the absolute value *R*_*s*_ after the second reduction may be due to slight uncertainties in the contact sizes in the Van der Pauw measurement and not to a degradation of the film, since the RRR value keeps the same as for the first reduction, and when normalised at 300 K, ρ/ρ_300 *K*_, shows a subsequent improvement (Figure 5a; Figure S7, Supporting Information).

The removal of oxygen anions in the reduced phase can also be tracked by the shrinkage of the *c*‐axis lattice parameter. Figure 5h shows the *c*‐axis lattice parameter as function of *T*_*onset*_. We find a striking ≈12% decrease in the lattice parameter along *c* from the parent perovskite phase to the complete topotactic oxygen deintercalation, and although a precise lattice parameter determination is hindered by the limited number of accessible Bragg reflections, our values are in agreement with those in literature for the SC IL PSNO_2_ phase.^[^ ^2^, ^3^ ^]^ This contraction along *c* leads to a decrease in the thickness of the film (estimated from the Scherrer equation), as depicted in Figure 5i, in agreement with STEM measurements as discussed below.

**Figure 6:**
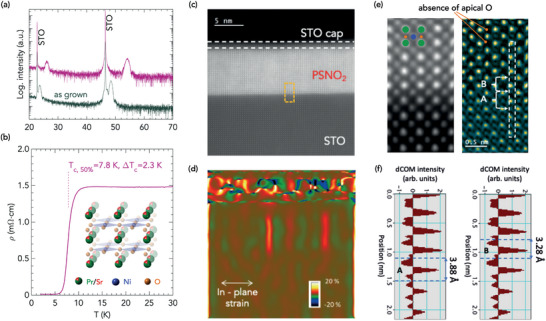
Structure of SC IL PSNO_2_ thin film. a) XRD θ − 2θ symmetric scans of a PSNO_3_ film as‐grown (grey) and after reduction (pink), showing reflections consistent with the IL tetragonal PSNO_2_ phase. The curves are vertically offset for clarity. b) Temperature dependent resistivity ρ(*T*) of the reduced sample, showing the SC transition at $T_{c,50\%}=7.8\;K$. (Inset: schematic of the crystal structure of the IL phase PSNO_2_ with Ni^1.2 +^ in a square‐planar environment (tetragonal space group P4/mmm)). c) Atomic‐resolution HAADF‐STEM image of the IL film, scale bar, 5 nm. d) Geometrical Phase Analysis of the in‐plane components of the strain tensor obtained from the STEM image in panel *c*. The red regions show possible Ruddlesden–Popper defects. e) Atomic‐resolution HAADF‐STEM image (left) from the region highlighted in panel *c* as a dashed orange rectangle (green circles indicate Pr/Sr atom sites; blue circles, Ni atom sites; and, orange circles, oxygen in the NiO_2_ planes); and, 4D‐STEM dCOM image (right) revealing the absence of apical oxygen anions. White arrows show positions between which distances are calculated in panel *f*. f) Calculated dCOM intensity profile across the PSNO_2_|STO interface along the atomic column highlighted in the 4D‐STEM dCOM image (panel *e*, right): oxygen‐oxygen distances of 3.88 Å in STO and 3.28 Å in PSNO_2_ are found (locations marked as A and B, respectively, in panel *e*, right). All the measurements were taken from the same sample.

The structural analysis of the IL phase was completed by STEM on an optimized IL PSNO_2_ film whose XRD pattern and temperature dependent resistivity are shown in **Figure** **6**a, b, showing a critical temperature of $T_{c,50\%}=7.8\;K$. Inset in Figure 6b depicts the schematic of the crystal structure of the IL phase, where the infinite NiO_2_ planes are separated only by Pr/Sr atoms, once the apical oxygen anions of the starting perovskite phase have been removed. The cross‐sectional STEM image of the IL film in Figure 6c shows a high‐quality infinite‐layer structure. And, the geometrical phase analysis (GPA) algorithm applied to that STEM image in Figure 6d reveals only a few possible Ruddlesden–Popper defects. Furthermore, 4D‐STEM divergence of center of mass (dCOM) image, approximating to a projected charge density image in Figure 6e confirms the removal of apical oxygen anions from the perovskite phase so that Pr/Sr planes alternate with NiO_2_ planes in the film. We also observed an abrupt oxygen–oxygen distance variation at the interface of the reduced film (Figure 6f), changing from ≈3.9 Å in the STO substrate (left panel, A) to ≈3.3 Å in the IL PSNO_2_ film (right panel, B), which confirms the topotactic transformation at the interface. Moreover, the out‐of‐plane lattice parameter estimated from the GPA analysis depicted in Figure S14 (Supporting Information) corresponds to a decrease of 15% related to the STO lattice parameter (3.91 Å), closely matching the values determined for the fully reduced sample by X‐ray diffraction in Figure 5i.

The present work opens up the prospect of experimentally studying the structure of the substrate‐nickelate interface at atomic scale, which goes beyond the scope of this work. Specifically, questions arise about possible alternative atomic interfaces (see Figure S15, Supporting Information), different to that previously reported,^[^ ^61^ ^]^ and whether the topotactic transformation might result in interface reconstructions revealed by first principles calculations for Nd‐nickelate^[^ ^62^ ^]^ or La‐nickelate.^[^ ^63^ ^]^

We finally mention that attempts to obtain SC films from uncut samples were always unproductive and a deeper understanding of the topotactic process is needed to explain this empirical observation. Immediately following unsealing of the ampoule, the uncut film shows XRD patterns or temperature dependence of resistivity typical of a reduced phase, but it readily reoxidizes in less than 24 h even when stored in a glovebox under nitrogen atmosphere or under vacuum (see Section S11 and Figures S16–S18, Supporting Information).

## 3 Conclusion

In summary, we have accomplished the synthesis of SC ‐ IL praseodymium nickelate thin films. Our results highlight the importance of the combined optimization of both steps of the process: perovskite growth, with control over cation stoichiometry, and topochemical reduction. Starting from nearly stoichiometric perovskite films with minimal defect densities, the linear‐in‐temperature resistivity of intermediate reduced films can be used as a proxy to assess the performance of subsequent reduction processes. These results contribute towards the goal of yielding high quality superconducting nickelate samples, still scarce to date, to enable further experimental progress in this field. Furthermore, better understanding of the topochemical aspects of the reduction process is a critical issue for exploring this new class of superconductors and could push forward experimental research on the field.

## 4 Experimental Section

### Pulsed Laser Deposition and CaH_2_ Reduction

Perovskite nickelate thin films were grown in a PLD system that utilizes a KrF excimer laser (248 nm) focused onto the target. The laser pulse rate was fixed at 4 Hz. During the growth, oxygen was supplied in the PLD chamber yielding a background pressure up to 0.5 mbar. The laser fluence was varied between 1.2 and 2.5 J cm^−2^, with a laser spot size of 2 ± 0.2 mm^2^. The substrate temperature was set in the range 570 – 675 °C. These parameters were optimized to produce high‐quality epitaxial films as discussed in the text. The films were cooled down to room temperature at a rate of 5 °C min^−1^ at the growth pressure. Single crystals of (001)SrTiO_3_ (STO) were used as substrates. Prior to the growth, they were etched in buffered HF and annealed at 1000 °C for 3 h to obtain stepped surfaces. The PLD target was sintered from a mixture of Pr_2_O_3_ (99.99%), Nickel(II) oxide (99.99%) and SrCO_3_ with controlled cation stoichiometry ([Ni]/([Pr]+[Sr]) = 1.1; [Pr]/[Sr] = 4) by a solid state reaction. These mixtures were ground in an agate mortar and, after initial decarbonation at 1200 °C for 12 h, pressed into pellets, and heated in a box furnace at 1300 °C for 24 h. To get high‐density PLD targets, the powders were reground and repressed, and then fired at 1300 °C for further 24 h.

Reduction of the perovskite phase into the IL phase was carried out in evacuated glass tubes. The tubes were filled with 0.1 g of CaH_2_ powder in an N_2_‐filled glovebox. Samples were wrapped in aluminum foil and inserted into the glass tubes. The samples were separated from the CaH_2_ powder (Alfa Aesar A16242) by means of a lump of glass wool (see Figure S19, Supporting Information). The tubes were evacuated by means of a rotary pump (<10^−3^ mbar) and sealed. A horizontal tube furnace, ventilated with N_2_ for increased temperature homogeneity was used for the process, with temperature accuracy ±1 °C. The reference temperature was measured at the center of the tube furnace, where the sealed ampoule was placed. Heating and cooling rates in the oven were 5 °C min^−1^.

### X‐Ray Diffraction

XRD θ/2θ scans of the films were performed in a four‐circle diffractometer (Cu source, Ge(220) 2‐bounce incident beam monochromator). The lattice parameters were calculated from Gaussian fits of the (001) and (002) XRD peaks (indices with respect to the pseudocubic unit cell) and extrapolated against cos ^2^θ/sin θ to θ = 90° to reduce systematic errors. The thickness of the reduced films was estimated from the width of the (002) XRD reflection through the Scherrer equation with a constant *K* = 1.06 fitted for the samples. Scans around asymmetrical reflections of the films were transformed into Reciprocal Space Maps (RSMs).

### X‐Ray Photoelectron Spectroscopy

XPS measurements (in situ and ex situ) were performed using a Mg Kα source (1253.6 eV, 20 mA, 15 kV). Survey spectra were acquired with a pass energy of 60 eV and detailed spectra with a pass energy of 20 eV in the energy analyzer. All spectra were measured in normal emission. XPS data were processed with the CasaXPS software.

### Electrical Transport

Transport measurements were performed in a Physical Property Measurement System (PPMS, Quantum Design). Four‐point resistivity measurements were performed in a Van der Pauw geometry by means of wire‐bonded Au wires. Temperature‐dependent Hall coefficients were calculated from linear fits of antisymmetrized field sweeps up to 9 T.

### Transmission Electron Microscopy

The cross‐sectional lamellae for Transmission Electron Microscopy were prepared using a Focused Ion Beam (FIB) technique at Centre de Nanosciences et de Nanotechnologies (C2N), University Paris‐Saclay, France. Prior to FIB lamellae preparation, around 20–30 nm of amorphous carbon was deposited on top of the samples for protection. The high‐angle annular dark‐field (HAADF) imaging and 4D‐STEM was carried out in a NION UltraSTEM 200 C3/C5‐corrected scanning transmission electron microscope (STEM). The experiments were done at 200 keV with a probe current of ≈12 pA and convergence semi‐angles of 23 mrad. A MerlinEM (Quantum Detectors Ltd) in a 4 × 1 configuration (1024 × 256) had been installed on a Gatan ENFINA spectrometer mounted on the microscope.^[^ ^64^ ^]^ For 4D‐STEM, the electron energy loss spectroscopy (EELS) spectrometer was set into non‐energy dispersive trajectories and six‐bit detector mode that gave a diffraction pattern with a good signal to noise ratio without compromising much on the scanning speed was used. The geometrical phase analysis (GPA)^[^ ^65^ ^]^ had been done choosing the STO substrate with 3.91 Å as a reference parameter. The lattice parameters of the PSNO_2_ were estimated by averaging the GPA maps over square areas of ≈ 50 (in‐plane) × 50 (out‐of‐plane) nm giving a strain accuracy determination better than 1%, that is, better than 0.04 Å for the lattice parameters. Such an approach had been previously employed to accurately determine the *c*‐axis variation in an apical oxygen ordered nickelate thin‐film on an STO substrate.^[^ ^66^ ^]^ The EELS spectra were obtained using the full 4 × 1 configuration and the 4D‐STEM by selecting only one of the chips (256 × 256 pixels). The element maps were done by integrating the core EELS edge‐signal of the respective elements and mapping them in the spectrum image.

## Conflict of Interest

The authors declare no conflict of interest.

## Author Contributions

A.G.L. designed and performed the experiments (growth, XRD, XPS, topochemical reductions, electrical transport), processed and analyzed the data, produced the graphics and wrote the manuscript. A.R. and A.G. designed and performed electron microscopy experiments, processed and analyzed the data. D.Z. and F.G. performed complementary experiments. L.D. and C.G. designed and implemented the experimental setup for sealing the glass ampoules used in the reduction process. L.I. initiated the optimization (growth, XRD, topochemical reduction, electrical transport) of nickelate samples at the beginning of the project and assisted in reduction experiments. M.B. proposed the project, provided equipement and acquired funding. A.G.L., A.R., D.Z., A.G., L.I. and M.B. discussed the data. All co‐authors reviewed the manuscript, and approved its final form.

## Supporting information

Supporting Information

## Data Availability

The data that support the findings of this study are available from the corresponding author upon reasonable request.
