# Supplementary material for: Toward Reliable Synthesis of Superconducting Infinite Layer Nickelate Thin Films by Topochemical Reduction
Source: Adv Sci (Weinh). 2024 Apr 18;11(24):2309092. doi: 10.1002/advs.202309092 (PMC11200026; doi:10.1002/advs.202309092)
Supplement: Supplementary file 1 — Supporting Information [file ADVS-11-2309092-s001.pdf]

ADVANCED SCIENCE
Open Access

Supporting Information

for *Adv. Sci.*, DOI 10.1002/advs.202309092

Toward Reliable Synthesis of Superconducting Infinite Layer Nickelate Thin Films by Topochemical Reduction

Araceli Gutiérrez-Llorente, Aravind Raji, Dongxin Zhang, Laurent Divay, Alexandre Gloter, Fernando Gallego, Christophe Galindo, Manuel Bibes and Lucía Iglesias**

Supplemental Material

Towards reliable synthesis of superconducting infinite layer nickelate thin films by topochemical reduction

Araceli Gutiérrez–Llorente,[1,2,*] Aravind Raji,[3,4] Dongxin Zhang,[2] Laurent Divay,[5] Alexandre Gloter,[3] Fernando Gallego,[2] Christophe Galindo,[5] Manuel Bibes,[2] and Lucía Iglesias[2,†]

[1] *Universidad Rey Juan Carlos, Escuela Superior de Ciencias Experimentales y Tecnología, Madrid 28933, Spain*
[2] *Laboratoire Albert Fert, CNRS, Thales, Université Paris Saclay, 91767 Palaiseau, France*
[3] *Université Paris Saclay, CNRS, Laboratoire de Physique des Solides, 91405 Orsay, France*
[4] *Synchrotron SOLEIL, L'Orme des Merisiers, BP 48 St Aubin, Gif sur Yvette, 91192, France*
[5] *Thales Research & Technology France, 91767 Palaiseau, France*

CONTENTS

1. Optimal growth of $PSNO_3$ films S2
2. Low-temperature resistivity of $PSNO_3$ films grown under non-optimal conditions S3
3. Cation stoichiometry of the $PSNO_3$ films by X-ray Photoelectron Spectroscopy S4
4. Resistivity of SC $PSNO_2$ films S7
5. XRD patterns of topochemically reduced films S8
6. Fits of the normal-state resistivity of SC $PSNO_2$ films S8
7. Reductions beyond the linear-in temperature resistivity of the normal state S9
8. Additional transport characterization of SC $PSNO_2$ film S10
9. Scanning transmission electron microscopy of SC $PSNO_2$ film S10
10. STEM-EELS element map of $PSNO_3/SrTiO_3$ interface S11
11. Topochemical reduction on uncut samples S12
12. Supplementary details on the topotactic reduction process S14

References S14

1. Optimal growth of $PSNO_3$ films

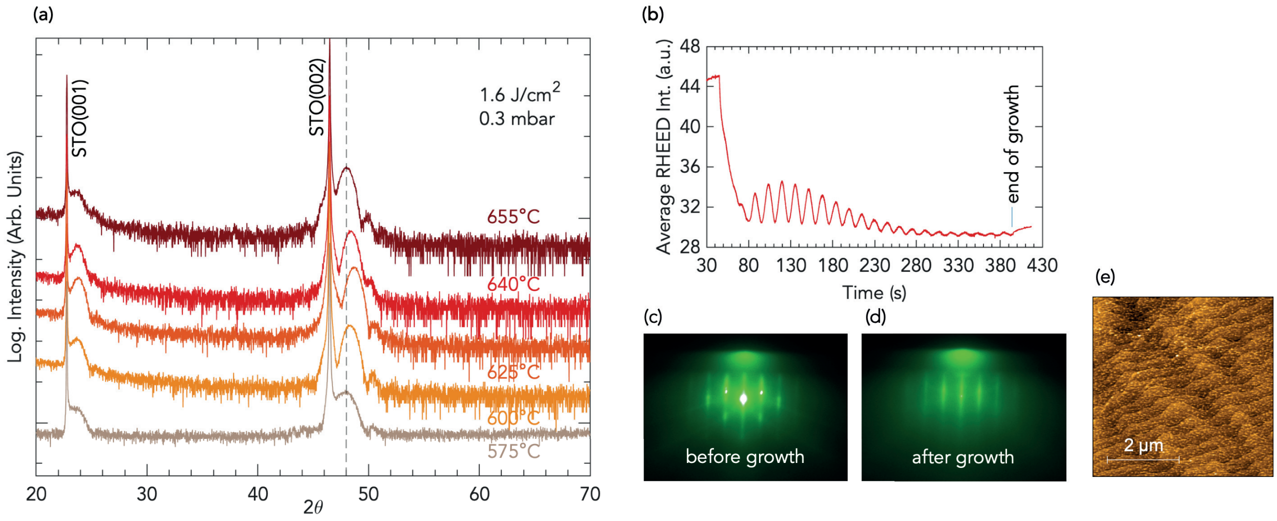

FIG. S1. **(a)** XRD symmetric $\theta - 2\theta$ scan patterns of $PSNO_3$ films grown on STO substrates at the optimized conditions of laser fluence 1.6 J/cm^2, oxygen pressure 0.3 mbar, and substrate temperatures ranging from 575 °C to 655°C. The dashed horizontal line is an estimate of c from the bulk lattice constant of PNO_3 strained to the STO substrate, and doping with Sr is expected to bring about a contraction of the unit cell. **(b)** RHEED intensity oscillations observed during the growth of a $PSNO_3$ film under optimal conditions of 1.6 J/cm^2, 640°C, 0.3 mbar. RHEED diffraction patterns taken **(c)** before, and **(d)** after the growth shown in panel (b). **(e)** AFM image of the as-grown film.

2. Low-temperature resistivity of $PSNO_3$ films grown under non-optimal conditions

Structural or composition disorder results in a decrease of the mean free path of the carriers, and quantum mechanical corrections to the low-temperature conductivity should be taken into account, since even in the weak-disorder limit the Boltzmann description $\rho(T) = \rho_0(T)$ is no longer valid.

We have fitted the experimental resistivity data at low-temperature to the temperature dependence[1]

$$\rho(T) = \frac{1}{\sigma_0 + a\ln(T)} + bT^2 \tag{S1}$$

where σ_0 is the residual conductivity due to scattering by defects, and is a measure of the degree of disorder; the term propotional to $\sim \ln$ gives the quantum correction of the conductivity; and, the term bT^2 accounts for the classical low-temperature dependence of the resistivity. Table S1 shows the fitting parameters to that behaviour. We observe higher degree of disorder in the film grown at higher laser fluence, with the minima in resistivity occurring at a highest temperature.

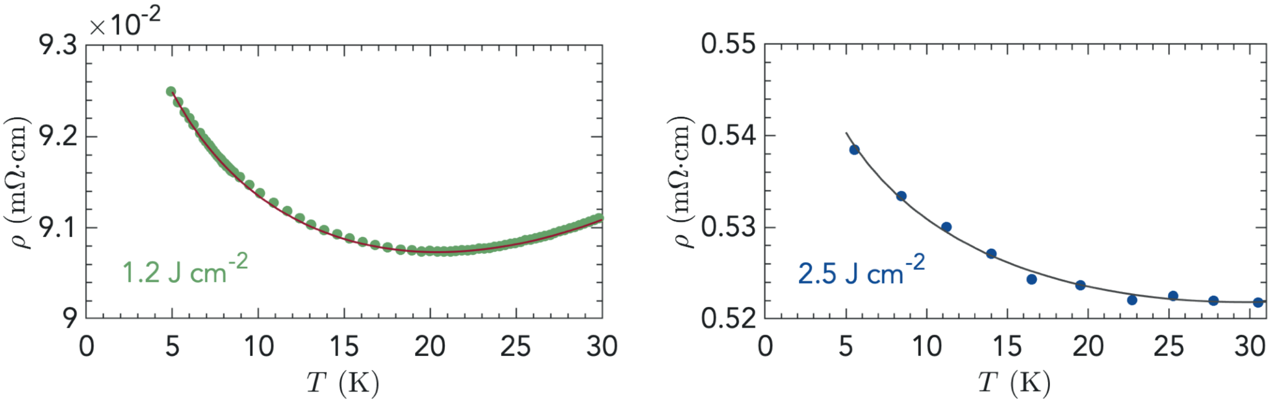

FIG. S2. Temperature dependence of resistivity,$\rho(T)$, at low temperatures in $PSNO_3$ films grown at 600 °C, 0.4 mbar and a laser fluence of 1.2 $\mathrm{J\,cm^{-2}}$ (left panel) and 2.5 $\mathrm{J\,cm^{-2}}$ (right panel). Solid circles are experimental data and solid lines are least-square fits of the experimental data to Eq.S1.

TABLE S1. Fitting parameters of experimental resistivity as a function of temperature to the Eq.S1 in the temperature range from 5 K to 30 K. Fits are shown in Fig.S2

laser fluence	T_{min} (K)	σ_0	a	b
1.2 $\mathrm{J\,cm^{-2}}$	10.8	10.47	0.22	$2.3 \cdot 10^{-6}$
2.5 $\mathrm{J\,cm^{-2}}$	30.5	1.77	0.05	$8.3 \cdot 10^{-6}$

These quantum-mechanical corrections have two contributions: localization of the wave function and electron-electron interaction, leading both of them to an increase of the resistivity as the temperature decreases with very similar dependence on temperature (either in 3D or 2D models).[2] Differentiating both contributions would require careful analysis of the field dependence of the low-temperature resistivity,[1] which is beyond the scope of this paper.

3. Cation stoichiometry of the PSNO$_3$ films by X-ray Photoelectron Spectroscopy

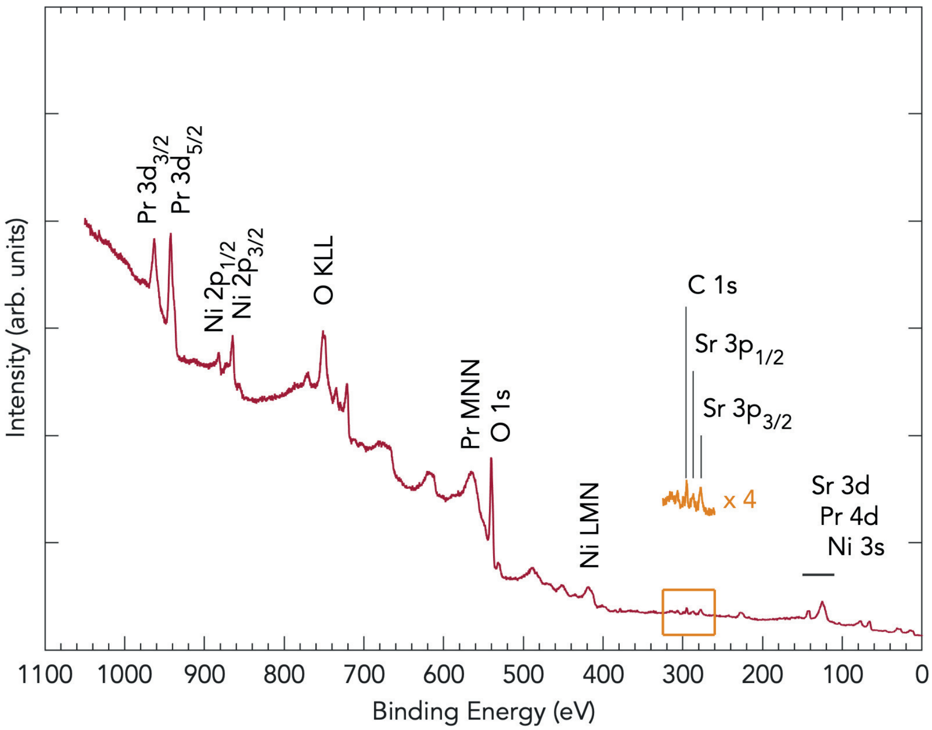

FIG. S3. Mg Kα-excited XPS survey spectrum performed at high pass energy (60 eV) on a perovskite SPNO$_3$ film showing the assignments of the peaks. Pr 3d and Ni 2p core-levels are used for quantification of the [Pr]/[Ni] ratio, as detailed below. Quantification of Sr is done from Sr 3p$_{3/2}$ since Sr 3p$_{1/2}$ overlaps the C 1s region, and Sr 3d core level overlaps Pr 4d and Ni 3s regions.

Quantitative XPS for the determination of the [Pr]/[Ni] ratio was derived from the area under the core-level and satellite peaks, Pr 3d and Ni 2p. The Pr 3d core level spectra consist of a spin-orbit split doublet with Pr $3d_{5/2}$ and Pr $3d_{3/2}$ components, at 3:2 area ratio, and Ni 2p core level spectra consist of a doublet Ni $2p_{3/2}$ and Ni $2p_{1/2}$, at 2:1 ratio, resolved in energy as illustrated in Fig.S4(a) and (b), respectively. Peak areas were obtained after subtraction of a Tougaard background extending over both components for each doublet.[3] To minimize impact of sample charging observed during data acquisition, samples were surrounded with conductive silver paste to provide surface conductive paths during measurements and ensure the best peak shape. Yet spectra are shown as function of relative binding energy (BE) having the position at the maximum envelope as the zero of the energy scale, since relative peak positions were observed to be stable, and we were not concerned with absolute values of BE but with quantitation using relative intensities.

Fig. S4(c, d) display peak models used for quantitative analysis of the [Pr]/[Ni] ratio through deconvolution into several component peaks. To obtain reasonably accurate fitting with meaningful results, the number of component peaks was kept to the minimum that enables an appropriate fit, and constraints were imposed across core-level spectra.[4,5] The spin-orbit splitting energies for Pr $3d_{5/2}$-Pr $3d_{3/2}$ and Ni $2p_{3/2}$-Ni $2p_{1/2}$ were $\Delta E_{\rm SO}(Pr) = 20.5$ eV$\pm$ 0.1 eV and $\Delta E_{\rm SO}(Ni) = 17.3$ eV$\pm$0.1 eV, respectively, in agreement with literature;[6,7] peak widths were constrained to have the same values in both components of a doublet within 10%, and peak area ratios between spin-split components were constrained to their theoretical ratio.

The Pr $3d_{5/2}$ spectrum was fitted with two components: the main peak, A_1^{Pr}, that can be attributed to $\mathrm{Pr}^{3+} - 4f^2$ final state, and its low binding energy satellite peak, B_1^{Pr}, Fig.S4(c). The separation between those components was kept at 4.6 eV $\pm$ 0.1 eV. The Pr $3d_{3/2}$ spectrum is more complicated. Besides the doublets related to the main component and its satellite, A_2^{Pr} and B_2^{Pr}, an additional extra structure appears at higher BE, that exists only in

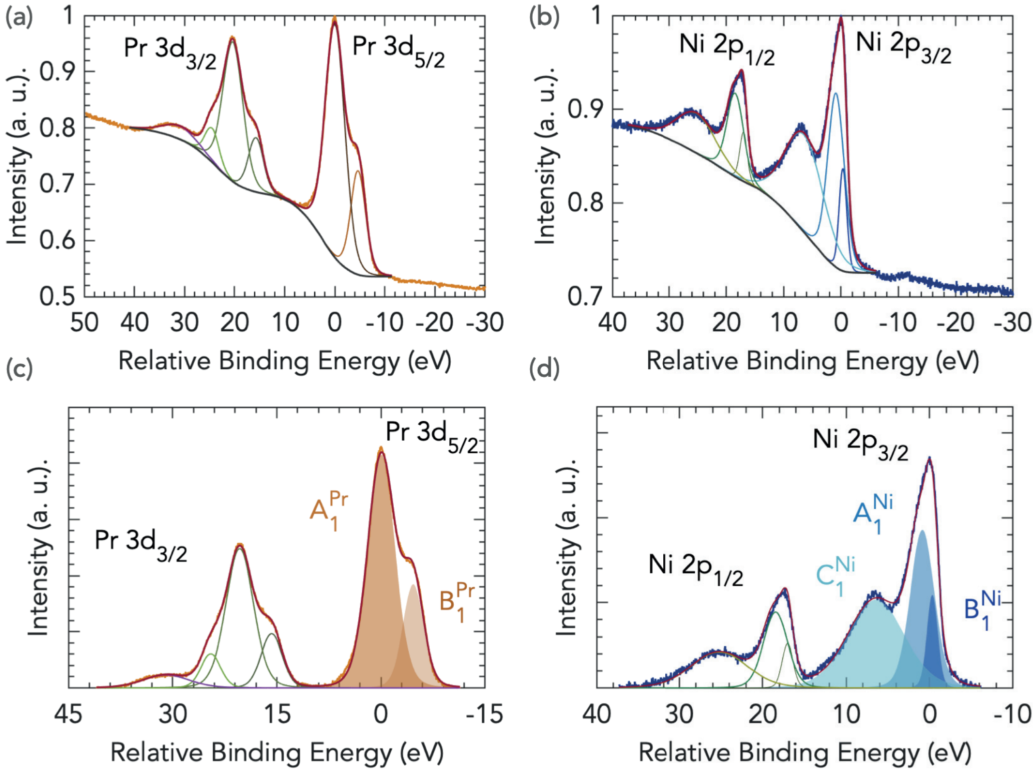

FIG. S4. **(a, b)** Examples of Pr 3d and Ni 2p doublets core-level XPS spectra, respectively, from a $PSNO_3$ film fitted with a Tougaard background extending over both components of each doublet. **(c, d)** Peak models used for the quantification of the [Pr]/[Ni] ratio. The components ((c) brown areas, Pr; (d) blue areas, Ni) and total fit envelope are shown.

Pr $3d_{3/2}$ (not in Pr $3d_{5/2}$) and has been assigned to a multiplet coupling effect.[6,8] Only Pr $3d_{5/2}$ spectrum was used for quantitative analysis in this work. No evidence of Pr^{4+} $(4f^1)$ was found in our XPS spectra, discerned in other Praseodymium compounds as a spectral feature between the Pr $3d_{5/2}$ and Pr $3d_{3/2}$ regions,[8–10] where our samples show no signal above the background. This is in agreement with a recent work on the role of Pr $4f$ orbitals on the electronic structures of the undoped and Sr-doped $PrNiO_2$ by density functional theory calculations,[11] where no sign of mixed valency for Pr was found and Pr $4f$ states were insulating without any hybridization channels near the Fermi energy. The Ni $2p_{3/2}$ spectrum was fitted with three components, A_1^{Ni} and B_1^{Ni}, whose separation was kept at 1.3 eV $\pm$ 0.1 eV, and a broad peak at higher BE, labelled C_1^{Ni} in Fig.S4(d).

Photoionization cross sections calculated by Scofield[12] lead to sensitivity factors of 30.72 for $Pr(3d_{5/2})$ and 13.92 for $Ni(2p_{3/2})$ that have been applied in Fig. 2 of the main text.

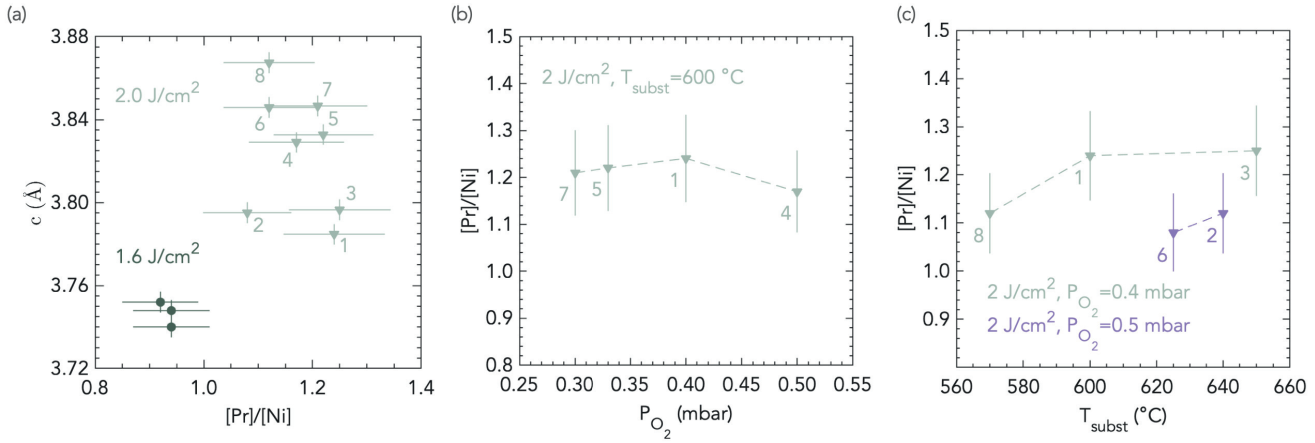

FIG. S5. Exploration of cation stoichiometry of films grown at high laser fluence (2 Jcm^{-2}). **(a)** c lattice parameter *vs* [Pr]/[Ni] ratio of $PSNO_3$ films grown at laser fluence 1.6 J cm^{-2}, 0.3 mbar and substrate temperatures of 625 °C or 640 °C, or under non optimal growth conditions of laser fluence 2 J cm^{-2} for different series of films, extracted from the fitted XPS spectra. **(b,c)**. [Pr]/[Ni] ratio of films as a function of oxygen pressure (b) and substrate temperature (c) for the series of films shown in (a). Error bars of 15% are applied to the [Pr]/[Ni ratio, which is the expected accuracy of XPS quantification for transition metal oxides.[5].

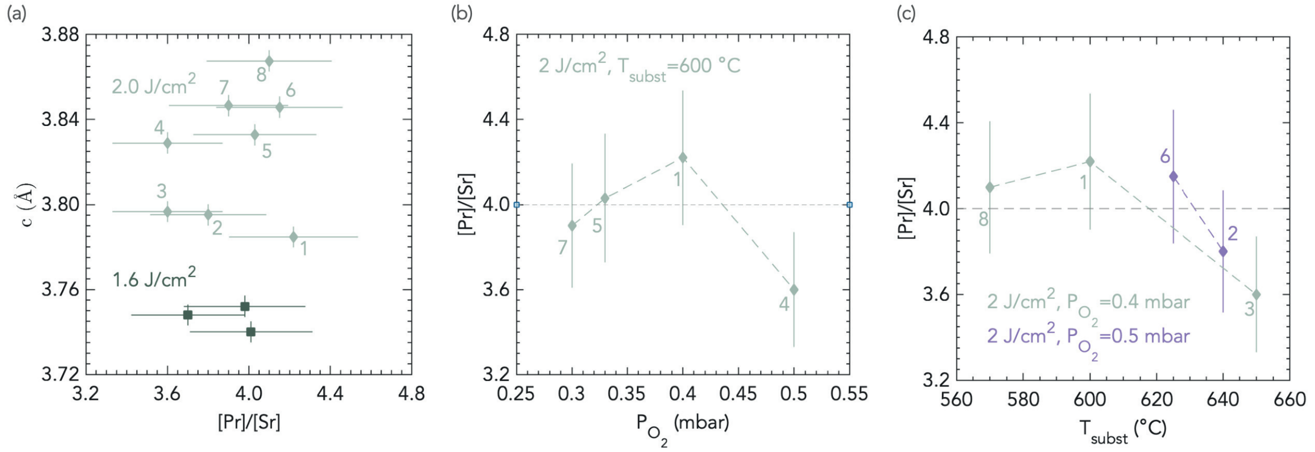

FIG. S6. Exploration of cation stoichiometry of films grown at high laser fluence (2 Jcm^{-2}). **(a)** c lattice parameter *vs* [Pr]/[Sr] ratio of $PSNO_3$ films grown at laser fluence 1.6 J cm^{-2}, 0.3 mbar and substrate temperatures of 625 °C or 640 °C, or under non optimal growth conditions of laser fluence 2 J cm^{-2} for different series of films, extracted from the fitted XPS spectra. **(b,c)**. [Pr]/[Sr] ratio of films as a function of oxygen pressure (b) and substrate temperature (c) for the series of films shown in (a). Error bars of 15% are applied to the [Pr]/[Sr] ratio in all the panels, which is the expected accuracy of XPS quantification for transition metal oxides.[5]

4. Resistivity of SC $PSNO_2$ films

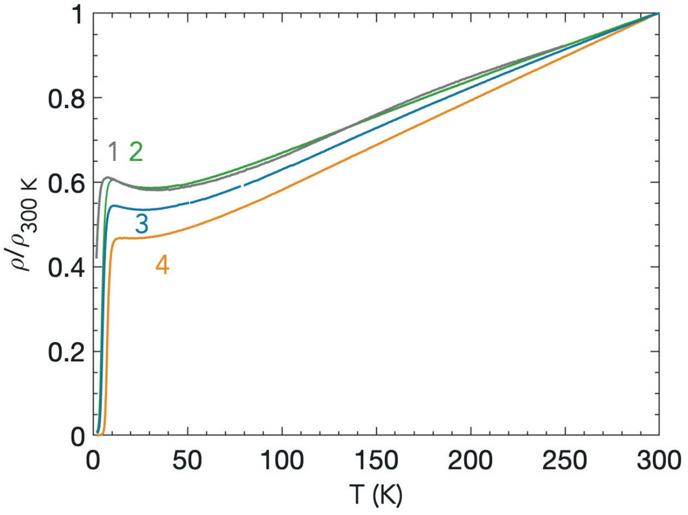

FIG. S7. Normalised resistivity in the range from 0 K to 300 K of samples analysed in Fig. 4 of the main manuscript. Color code as in the main figure.

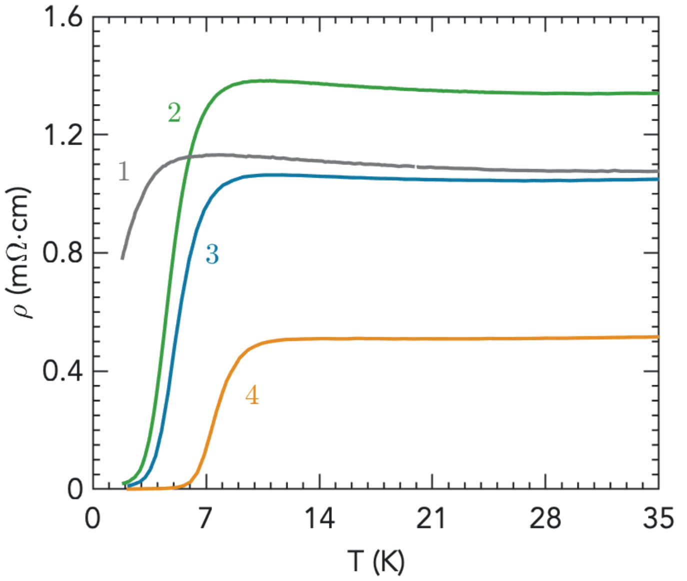

FIG. S8. Unnormalized resistivity of samples analysed in Fig. 4 of the main manuscript. Color code as in the main figure.

5. XRD patterns of topochemically reduced films

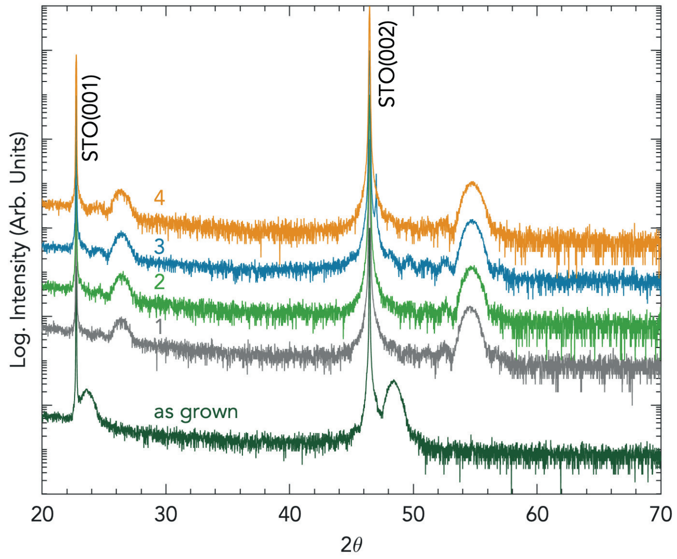

FIG. S9. XRD $\theta - 2\theta$ symmetric scans of the as-grown and reduced films after consecutive reduction processes carried out at 260°C for periods of (1) 150 min, and additional 30 min (2), 25 min (3), and 45 min (4), whose superconducting parameters are shown in Fig. 4 of the main text. The curves are vertically offset for clarity. Color code as in the main figure.

6. Fits of the normal-state resistivity of SC $PSNO_2$ films

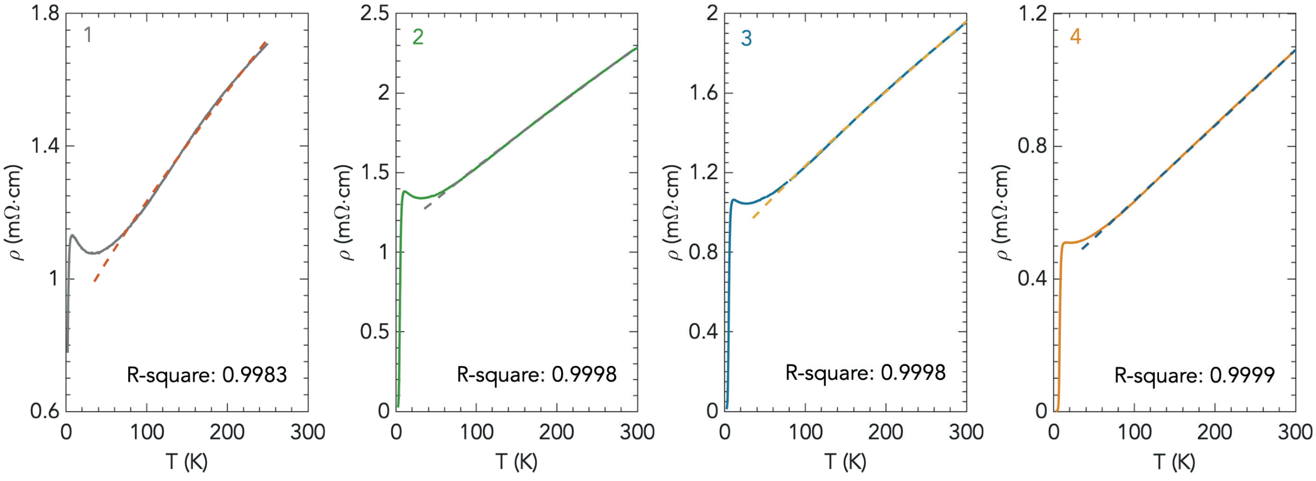

FIG. S10. Least-square fitting of the experimental data to $\rho(T) = \rho_{res} + AT^{\alpha}$ in the range from 300 K to 60 K over subsequent reductions.

7. Reductions beyond the linear-in temperature resistivity of the normal state

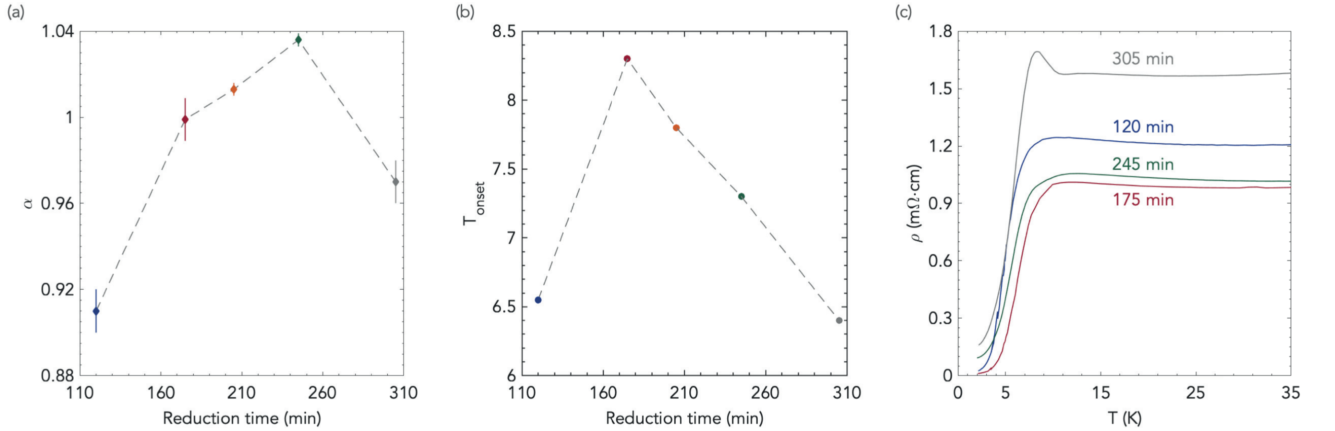

FIG. S11. (a) Exponent α in $\rho(T) = \rho_{res} + AT^{\alpha}$ of the fitting of the experimental data to $\rho(T) = \rho_{res} + AT^{\alpha}$ in the range from 300 K to 60 K over subsequent reductions. After 245 min of reduction a value of $\alpha \approx 1$ is observed. Reductions beyond that point decrease the onset temperature of the superconducting transition (b), and deteriorate the reduced phase, as shown in the temperature dependence of resistivity (c).

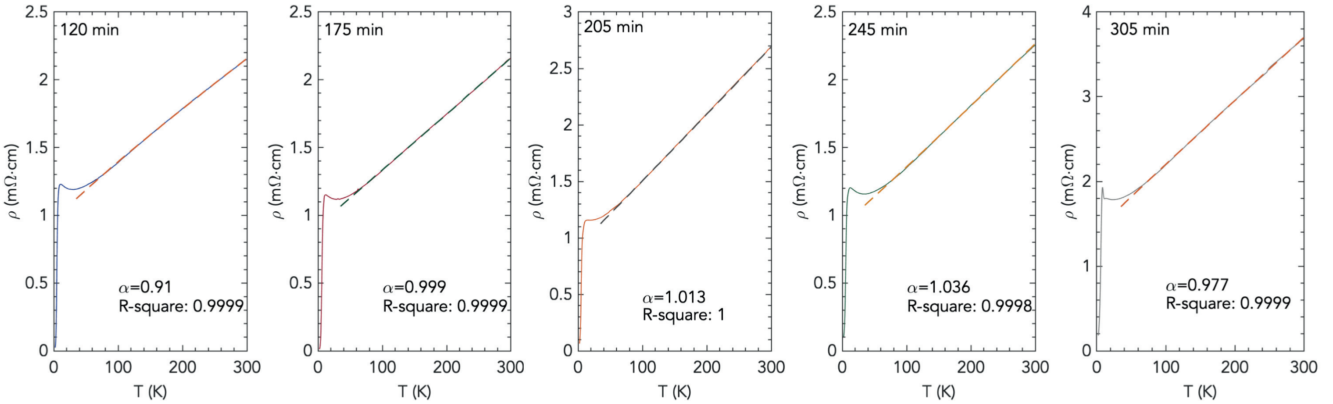

FIG. S12. Least-square fitting of the experimental data to $\rho(T) = \rho_{res} + AT^{\alpha}$ in the range from 300 K to 60 K over subsequent reductions. The value of the fitting parameter α as a function of the reduction time is shown in Fig.S11(a).

8. Additional transport characterization of SC $PSNO_2$ film

Hall resistivity is obtained by the usual antisymmetrization procedure, carried out between the positive and negative field sweeps in order to cancel out longitudinal resistivity (which is symmetric in the magnetic field).

Hall resistivity versus magnetic field for three different ranges of temperature is shown in Fig.S13 ((a,b) normal state; (c) mixed state). It leads to a Hall coefficient $R_H = (-1.4 \pm 0.03) \cdot 10^{-3}\mathrm{cm}^3\mathrm{C}^{-1}$ for $T/T_c = 13.2$ (Fig.S13(a)) and $R_H = (-8.4 \pm 0.3) \cdot 10^{-4}\mathrm{cm}^3\mathrm{C}^{-1}$ for $T/T_c = 3.3$ (Fig.S13(b)).

It is worth noting that typical values of the upper critical field for this system are much higher that the highest magnetic field applied in our experiments (see, for example, Wang et al., Science Advances, 9, 2023, Fig.1(E)).[13] Thus, the applied magnetic field can enter the superconductor (in the form of quantised flux lines or vortices), which is in mixed state. Moving vortices lead to dissipation in the mixed state of the superconductor, being associated with the appearance of a non-zero resistivity mixed state Hall effect. In the critical temperature region (Fig.S13,(c)) there is a normal-state contribution (linear in H) and a departure from linearity occurs in the magnetic field dependence of Hall resistivity at low fields. A detailed investigation of the magnetic field dependence of the mixed-state Hall resistivity should be carried out in samples patterned into a Hall bar configuration at various current densities and is beyond the scope of this work.

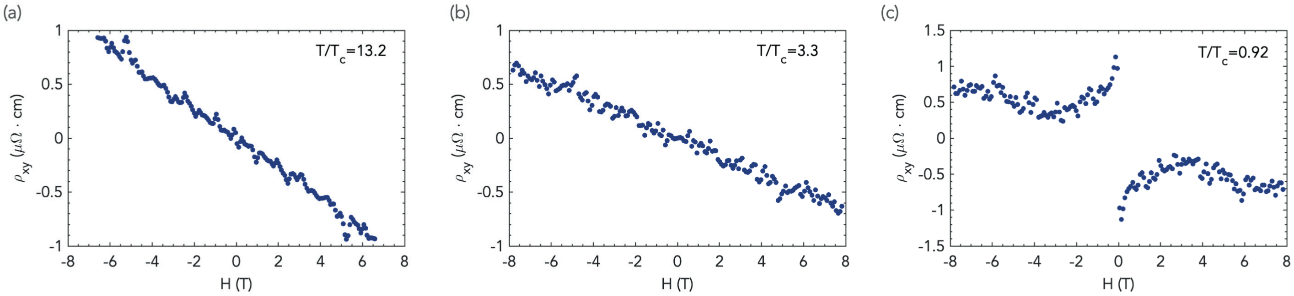

FIG. S13. Antisymmetrized Hall resistivity as a function of magnetic field at different temperatures for an unpatterned fully reduced SC film ($T_c = 7.6$ K, $\Delta T_c = 2.9$ K).

9. Scanning transmission electron microscopy of SC $PSNO_2$ film

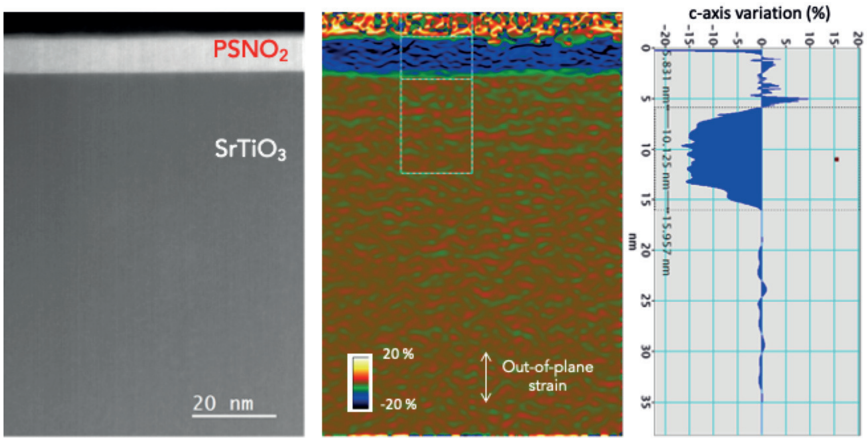

FIG. S14. Strain map along the out-of-plane direction (center panel) generated from the HAAF-STEM image (left panel) with GPA algorithm. It leads to an estimation of $c \approx 3.32$ Å for the out-of-plane parametter of the IL $PSNO_2$ film (decrease of $\approx 15\%$ related to the lattice parameter of the STO substrate).

10. STEM-EELS element map of $PSNO_3/SrTiO_3$ interface

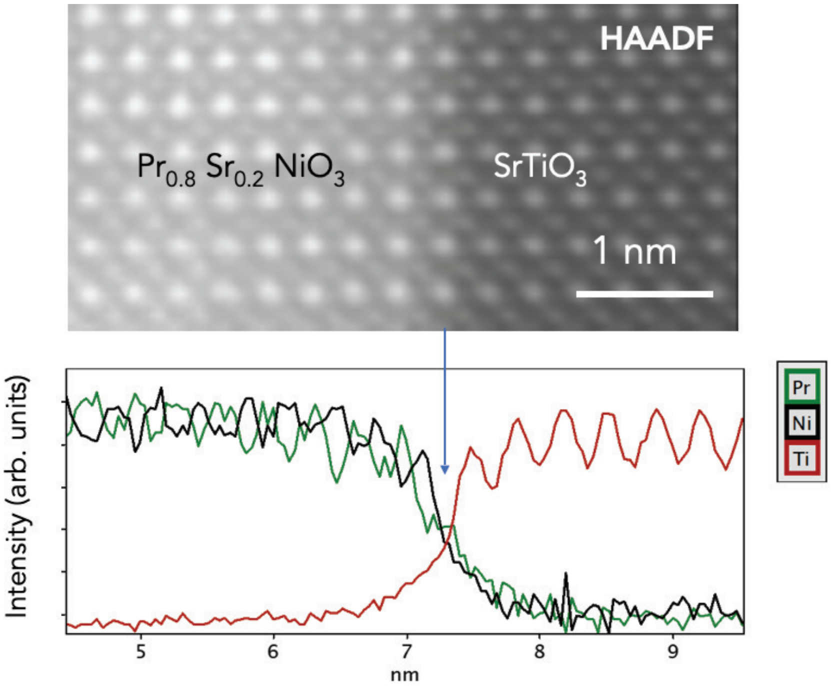

FIG. S15. STEM-EELS element map of a $PSNO_3$ thin film on the region near the interface. HAADF image of the region near the interface with $SrTiO_3$ (top panel) and corresponding element map of Ti-L, Pr-M, and Ni-L edges (bottom panel), showing the absence of the B-site intermixing (Ni/Ti) previously reported at both perovskite and infinite-layer nickelate-substrate interfaces.[14]

11. Topochemical reduction on uncut samples

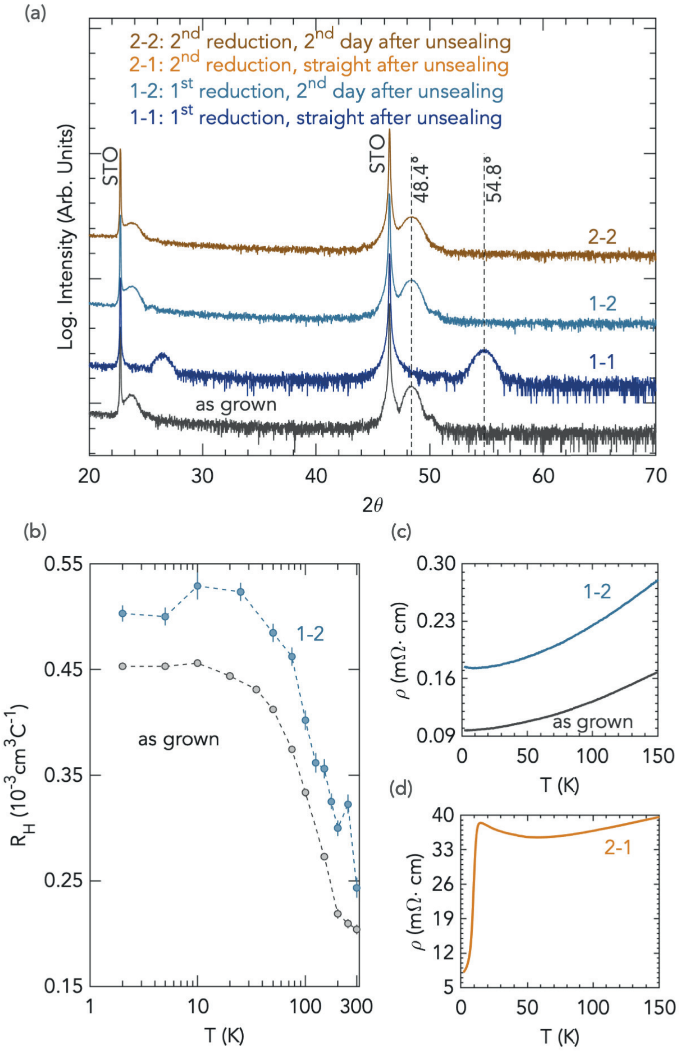

FIG. S16. Topochemical reduction performed on uncut samples of $PSNO_3$. **(a)** XRD θ-2θ symmetrical scans on a 5 × 5 mm^2 sample as-grown (bottom pattern, as reference); (1-1) after a topochemical reduction with CaH_2 at 260°C for 2h45min, and measured immediately after unsealing the ampoule, showing diffraction reflections consistent with a reduced phase; (1-2) measured the next day, showing the oxidised phase, with reflections at the same position than the as-grown sample; (2-2) after a subsequent reduction with CaH_2 at 260°C for additional 4h 15min, measured the second day after unsealing the ampoule. **(b)** R_H and **(c)** $\rho(T)$ of the sample 1-2 (first reduction step, measured 24 hours after the ampoule unsealing), confirmed the reoxidation observed in the XRD pattern in panel (a). $\rho(T)$ and R_H for the as-grown sample are shown as reference. **(d)** $\rho(T)$ from the sample (2-1), after second reduction step and measurement carried out right after unsealing the ampoule, showing a SC transition at low temperature consistent with a reduced phase, while the next day the sample has reoxidised as shown in XRD pattern (2-2) in panel (a). Error bars indicate the 1σ uncertainties of the fits.

Attempts to obtain SC films from uncut samples were always unproductive. Immediately following unsealing of the ampoule, the uncut film shows XRD patterns or temperature dependence of resistivity typical of a reduced phase, but it readily reoxidizes in less than 24 hours even when stored in a glovebox under nitrogen atmosphere or under vacuum. This swift reoxidation prevents us from measuring different properties after a reduction step, Fig.S16 Remarkably, once that sample is cut and the reduction is carried out on one of the resulting pieces, we did not observe reoxidation after successive annealings, as shown in Fig.S17. Previous works report perovskite films are cut in half before being reduced to the infinite-layer phase,[15–17] regardless of the size of the substrate.[18] It is worth

noting that reoxidation observed on the uncut reduced samples is not avoided by a STO capping layer, while no reoxidation on capped SC films that have been previously cut was detected within the course of several weeks after reduction (Fig.S18).

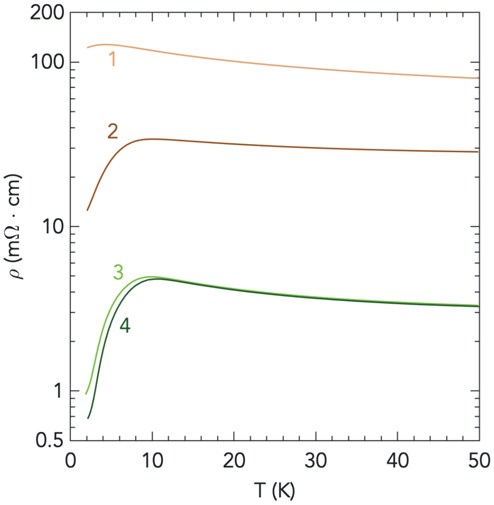

FIG. S17. After unproductive reduction attempts, detailed in Fig. S16, the sample was oxidised in flowing O_2 gas (atmospheric pressure) at 680°C for 12 hours, and cut into four pieces. Incremental reduction treatments on one of those pieces give rise to a SC transition that improves over successive reduction steps, although a zero-resistance state is not achieved, likely due to some degradation of its crystalline properties after several cycles of reduction/reoxidation that it underwent earlier.

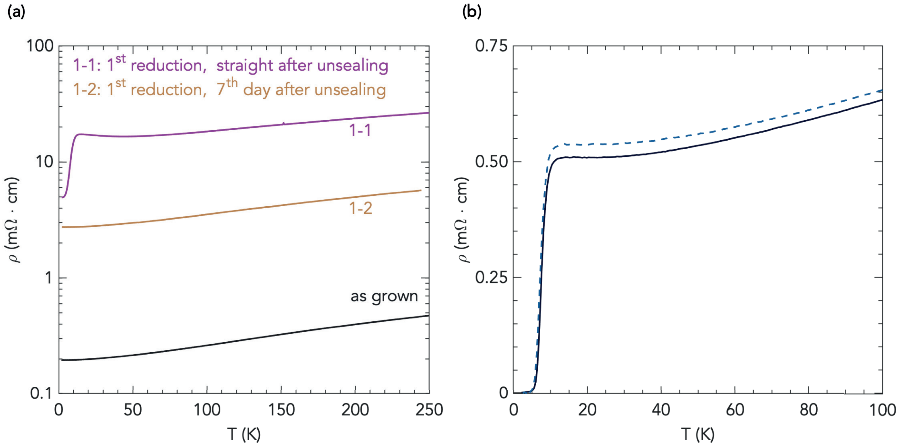

FIG. S18. Reoxidation of the uncut reduced samples is not avoided by an STO capping layer. **(a)** $\rho(T)$ of an uncut film with an STO capping layer immediately after unsealing the ampoule (1-1 plot), and 7 days later (1-2 plot), showing reoxidation. **(b)** No significant change to $\rho(T)$ of SC cut capped samples exposed to the air for up to several weeks is found. The dashed line plots the resistivity of a cut, capped sample 8 weeks after that plotted in solid line.

12. Supplementary details on the topotactic reduction process

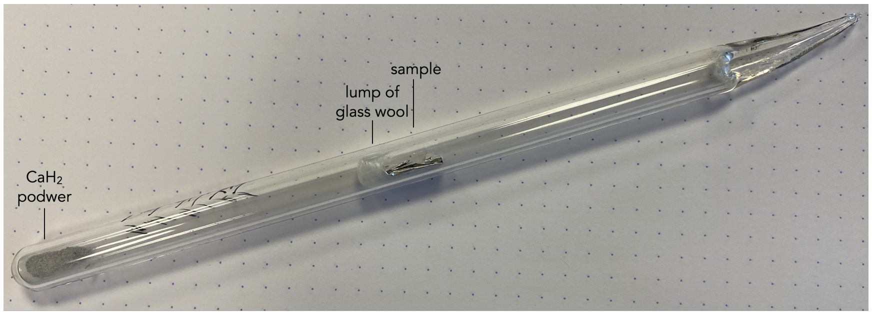

FIG. S19. Picture of a sealed tube used for topotactic reduction. The sample is wrapped in aluminum foil and separated from the CaH_2 powder by a lump of glass wool. The distance between the sample and the powder is kept constant for increasing reproducibility among reductions.

[*] araceli.gutierrez@urjc.es
[†] lucia.iglesias@cnrs-thales.fr
[1] G. Herranz, F. Saanchez, J. Fontcuberta, V. Laukhin, and J. Galibert, Physical Review B **72** (2005), 10.1103/PhysRevB.72.014457.
[2] P. A. Lee and T. V. Ramakrishnan, Reviews of Modern Physics **57** (1985), 10.1103/RevModPhys.57.287.
[3] S. Tougaard, Journal of Vacuum Science Technology A **39** (2021), 10.1116/6.0000661.
[4] A. G. Shard, Journal Vacuum Science Technology A **38** (2020), 10.1116/1.5141395.
[5] C. R. Brundle and B. V. Crist, Journal Vacuum Science Technology A **38** (2020), 10.1116/1.5143897.
[6] H. Ogasawara, A. Kotani, R. Potze, G. A. Sawatzky, and B. Thole, Physical REview B **44**, 5465 (1991).
[7] Z. Fu, J. Hu, W. Hu, S. Yang, and Y. Luo, Applied Surface Science **441**, 1048 (2018).
[8] S. Dash, T. Morita, K. Kurokawa, Y. Matsuzawa, N. L. Saini, N. Yamamoto, J. Kajitani, R. Higashinaka, T. D. Matsuda, Y. Aoki, and T. Mizokawa, Physical Review B **98** (2018), 10.1103/PhysRevB.98.144501.
[9] A. Yaremchenko, S. Patricio, and J. Frade, Journal of Power Sources **245**, 557 (2014).
[10] J. Gurgul, M. T. Rinke, I. Schellenberg, and R. Pöttgen, Solid State Science **17**, 122 (2013).
[11] X. Liao, M. R. Norman, and H. Park, Physical Review B **107** (2023), 10.1103/PhysRevB.107.165153.
[12] J. H. Scofield, Journal of Electron Spectroscopy and Related Phenomena **8**, 129 (1973).
[13] B. Y. Wang, T. C. Wang, Y. T. Hsu, M. Osada, K. Lee, C. Jia, C. Duffy, D. Li, J. Fowlie, M. R. Beasley, T. P. Devereaux, I. R. Fisher, N. E. Hussey, and H. Y. Hwang, Science Advances **9** (2023), 10.1126/sciadv.adf6655.
[14] B. H. Goodge, B. Geisler, K. Lee, M. Osada, B. Y. Wang, D. Li, H. Y. Hwang, R. Pentcheva, and L. F. Kourkoutis, Nature Materials **22** (2023), 10.1038/s41563-023-01510-7.
[15] D. F. Li, K. Lee, B. Y. Wang, M. Osada, S. Crossley, H. R. Lee, Y. Cui, Y. Hikita, and H. Y. Hwang, Nature **572** (2019), 10.1038/s41586-019-1496-5.
[16] K. Lee, B. H. Goodge, D. Li, M. Osada, B. Y. Wang, Y. Cui, L. F. Kourkoutis, and H. Y. Hwang, APL Materials **8** (2020), 10.1063/5.0005103.
[17] K. Lee, B. Y. Wang, M. Osada, B. H. Goodge, T. C. Wang, Y. Lee, S. Harvey, W. J. Kim, Y. Yu, C. Murthy, S. Raghu, L. F. Kourkoutis, and H. Y. Hwang, Nature **619** (2023), 10.1038/s41586-023-06129-x.
[18] J. Fowlie, M. Hadjimichael, M. M. Martins, D. Li, M. Osada, B. Y. Wang, K. Lee, Y. Lee, Z. Salman, T. Prokscha, J. M. Triscone, H. Y. Hwang, and A. Suter, Nature Physics **18** (2022), 10.1038/s41567-022-01684-y.
